# Towards Bright Single-Photon Emission in Elliptical Micropillars

**DOI:** 10.3390/nano13091572

**Published:** 2023-05-08

**Authors:** Aidar Galimov, Michail Bobrov, Maxim Rakhlin, Yuriy Serov, Dmitrii Kazanov, Alexey Veretennikov, Grigory Klimko, Sergey Sorokin, Irina Sedova, Nikolai Maleev, Yuriy Zadiranov, Marina Kulagina, Yulia Guseva, Daryia Berezina, Ekaterina Nikitina, Alexey Toropov

**Affiliations:** 1 Ioffe Institute, Politekhnicheskaya St. 26, 194021 St. Petersburg, Russia; aidar-34-dom94@yandex.ru (A.G.); toropov@beam.ioffe.ru (A.T.); 2 Laboratory of Nanoelectronics, St. Petersburg Academic University, 194021 St. Petersburg, Russia

**Keywords:** single-photon source, elliptical micropillars, quantum dots, optical modes

## Abstract

In recent years, single-photon sources (SPSs) based on the emission of a single semiconductor quantum dot (QD) have been actively developed. While the purity and indistinguishability of single photons are already close to ideal values, the high brightness of SPSs remains a challenge. The widely used resonant excitation with cross-polarization filtering usually leads to at least a two-fold reduction in the single-photon counts rate, since single-photon emission is usually unpolarized, or its polarization state is close to that of the exciting laser. One of the solutions is the use of polarization-selective microcavities, which allows one to redirect most of the QD emission to a specific polarization determined by the optical mode of the microcavity. In the present work, elliptical micropillars with distributed Bragg reflectors are investigated theoretically and experimentally as a promising design of such polarization-selective microcavities. The impact of ellipticity, ellipse area and verticality of the side walls on the splitting of the optical fundamental mode is investigated. The study of the near-field pattern allows us to detect the presence of higher-order optical modes, which are classified theoretically. The possibility of obtaining strongly polarized single-photon QD radiation associated with the short-wavelength fundamental cavity mode is shown.

## 1. Introduction

The development of highly efficient and perfectly controlled building blocks for quantum technologies is becoming an increasingly important topic. Efficient sources of indistinguishable single photons are one such building block [1,2]. Nonlinear spontaneous parametric down-conversion (SPDC) sources have long been the workhorse for generating single photons with high indistinguishability [3,4]. Despite these advances, SPDCs have a probabilistic nature of single-photon generation and can only operate in a low-brightness mode [5], which limits the implementation of scalable quantum computing and quantum communication schemes.

In contrast, sources based on two-level quantum emitters produce single photons during the lifetime of spontaneous radiative decay and emit only one photon at a time, which makes it possible to emit photons “on demand” [6,7]. Atoms [8,9], ions [10], colloidal quantum dots (QDs) [11], and impurity centers in crystals [12,13] can be used as a single quantum emitter. Self-assembled semiconductor QDs, however, are considered one of the most promising candidates due to a combination of unique properties. Compared to other systems, semiconductor QDs integrate naturally into the solid environment and thus do not require complicated vacuum systems and optical trapping setups. To date, various systems of semiconductor materials, including nitrides [14] and II–VI compounds [15], are used to create single-photon sources (SPSs). The choice of certain material systems makes it possible to obtain single photons in the desired spectral range, and the systems mentioned above allows one to create sources of single photons operating close to room temperature. Nevertheless, the technological difficulties of the epitaxial growth of structures in these systems significantly limit the achievable quality of SPS. In this regard, the InAs/GaAs material system seems to be the most promising, for which well-established growth techniques, such as molecular beam epitaxy (MBE) or metalorganic vapor phase epitaxy, offer the possibility to realize a monolithic growth procedure that provides monolayer accuracy and the designed emission wavelength. Moreover, InAs/GaAs self-assembled QDs have a high overall quantum efficiency of emission (more than 97%) [16].

However, the high contrast of refractive indices at the semiconductor–air interface limits the collection efficiency of QD emission to only a few percent. For the efficient collection of single photons, the idea of a photonic nanoantenna in the form of a cylindrical waveguide tapering towards the apex and providing a strong coupling with the fundamental HE${}_{11}$ mode was proposed [17]. Correctly chosen parameters of the photonic nanoantenna (base diameter, refractive index, and height) make it possible to increase the collection efficiency of QD emission up to 90% [18]. QDs in this design can emit in a wide spectral range and do not require spectral alignment of the emission line and the cavity mode. However, the drawback of the broadband approach is the presence of phonon sidebands and the possible appearance of undesirable additional emission lines of other QDs [19]. Another disadvantage is a significant decrease in the indistinguishability of single photons caused by the spatial proximity of the QD to the fluctuating charge medium of the semiconductor–air surfaces [20].

A more advanced method to improve the extraction efficiency is placing a QD in a resonator cavity to increase the local density of electromagnetic modes and accelerate its spontaneous emission into a given optical mode due to the Purcell effect [21]. To date, encouraging progress has been made in this area, with the demonstrations of very high extraction efficiency by introducing a QD into a photonic crystal cavity [22,23], a bullseye microcavity [24], and a micropillar cavity [25]. A micropillar cavity with a QD in a vertical λ cavity sandwiched between two distributed Bragg reflectors (DBRs) is by far the most successful design. Impressive brightness, purity and indistinguishability of single photons were obtained by resonant excitation of a QD in rotationally symmetric micropillars [26,27,28,29,30]. Resonant excitation by a π-pulse makes it possible to obtain the maximum pumping efficiency due to the complete population inversion of the QD transition state. The minimum temporal jitter between the acts of excitation and emission allows one to realize the best degree of single-photon indistinguishability in comparison with nonresonant pumping methods.

However, the method of cross-polarization laser filtering widely used with resonant excitation limits the maximum achievable efficiency to at least a factor of two since the light is projected in a single polarization. Alternative methods of excitation do not provide significant improvements. For example, in a two-color resonant excitation scheme [31], approximately 75% of the generated single photons are lost during the filtering stage. Off-resonant phonon-assisted excitation [32,33] reduces the QD population inversion and limits the achievable indistinguishability of single photons due to the participation of phonons.

One of the possible solutions is the use of elliptical microresonators [34]. The asymmetric microresonator is designed in such a way as to remove the spectral degeneracy of two orthogonally polarized V and H cavity modes, aligned parallel to the minor and major axes of the elliptical cross-section [35]. Single QDs embedded in such an elliptical micropillar demonstrated polarization-dependent Purcell enhancement, with single photons predominantly generated in one polarized state [36]. It was found that the degree of single-photon polarization in an elliptical microcavity depends on many factors, such as the cavity linewidth, spectral modes splitting, and the Purcell factor [37].

The first single-photon emission in elliptical microcavities was implemented using non-resonant excitation, which limited the indistinguishability, purity, and brightness of a single photon source [38,39]. Recently, H. Wang et al. demonstrated an SPS based on an elliptical microresonator with a brightness of 60% and indistinguishability of single photons of 0.975 [40]. The elliptical microresonator has the potential for further efficiency enhancement and is very promising for creating a commercial bright SPS due to its relatively simple design.

In this paper, we investigated the impact of the microcavity geometry on the single photon efficiency, considering the influence of its size and ellipticity. It turned out that one of the most important geometrical features is the critical sensitivity to the verticality of the side walls of the microresonator. The performed theoretical calculations of the optical mode parameters were verified by comparison with the experimentally measured reflection spectra and near-field luminescence patterns of various microcavities that revealed the presence of higher-order optical modes. The QD coupled to the fundamental linearly polarized optical cavity mode with the shorter wavelength was studied. With this approach, the energy of the exciting photons, aligned with the longer wavelength mode, turns out to be less than the energy of the QD transition. As far as we know, this work experimentally demonstrates for the first time the fundamental possibility of operating in such a mode, which opens the way to the possibility of further increasing the brightness of SPSs.

## 2. Materials and Methods

### 2.1. Fabrication of the Structure

**Figure 1:**
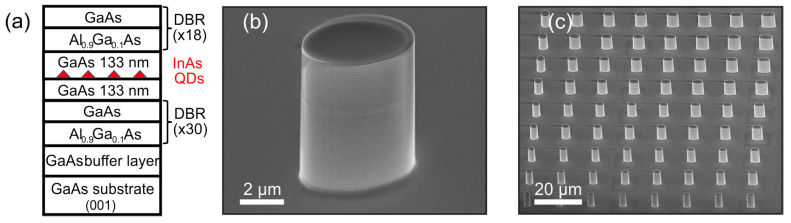
(**a**) MBE-grown semiconductor heterostructure consisting of 30(18) DBR layers and a GaAs λ-cavity with embedded self-assembled InAs QDs. (**b**) SEM image of a single elliptical micropillar and (**c**) a regular array of such micropillars, taken with a scanning electron microscope at a 45 degree angle. The height of the micropillars is 7.1 μm.

The heterostructure studied in this work was grown by molecular beam epitaxy on a GaAs:Si (001) substrate with a GaAs buffer layer 500 nm thick (Figure 1a). Over the buffer layer, 30(18) pairs of λ/4 Al${}_{0.9}$Ga${}_{0.1}$As/GaAs layers were grown, which formed the lower (upper) distributed Bragg reflectors. InAs QDs placed in a λ-cavity 266 nm thick were formed by the Stransky–Krastanov growth method with a surface density in the range of 10${}^{9}$–5 × 10${}^{9}$ cm${}^{-2}$.

Elliptical micropillars were fabricated by reactive ion–plasma etching in combination with contact photolithography (365 nm) based on a thick negative photoresist [41,42,43]. The etching technique has been optimized to produce highly vertical side walls with minimum roughness since the verticality of the sidewalls is especially important for precise control of the polarization mode splitting. A photomask was used with an array of round/elliptical cross-section elements of various sizes. This method makes it possible to create a set of elliptical micropillars with a variable aspect ratio within a single chip (Figure 1c) to obtain the required optical mode splitting. However, with this approach, the spatial coincidence of the QD position and the center of the micropillar is a random event, as well as the spectral coincidence of the QD emission line and the position of the optical resonance, which requires careful post selection of the micropillar structure with optimal characteristics based on the results of optical measurements. The geometric dimensions of the ellipses obtained after etching were determined from the scanning electron microscope (SEM) images. Figure 1b demonstrates a single elliptical micropillar with vertical sidewalls and excellent surface quality, obtained with an optimized fabrication process.

### 2.2. Optical Measurements

For optical measurements, the sample mounted in a helium cryostat was cooled to a temperature of 8 K. Laser radiation from a Ti–sapphire laser with a tunable wavelength and mode locking was focused on the apex of a selected micropillar using a lens with a numerical aperture of 0.42. The QD photoluminescence (PL) was collected with the same objective and analyzed using a triple grating spectrometer with a cooled CCD camera. Time-resolved measurements were carried out using superconducting single-photon detectors (Scontel) with a time resolution of 40 ps. To determine the purity of single photons, the Hanbury Brown–Twiss correlation scheme was used.

A halogen lamp coupled to a single-mode optical fiber with subsequent collimation makes it possible to measure the reflectance spectrum of a single micropillar. To obtain an image of optical modes, an array of microcavities was excited by a large laser spot.

### 2.3. Numerical Calculations

To simulate the emission intensity of a QD in elliptical microresonator, we used the full-vector three-dimensional finite-difference time-domain (FDTD) method, which is widely used for designing single-photon structures. This method is better suited for structures much larger than the wavelength under study. The FDTD simulation of the structure was carried out by embedding the microresonator in the air environment (refractive index n = 1) and the GaAs substrate at the bottom. Boundary conditions were set by adding a perfectly matched layer (PML) around the computational domain of the microcavity structure to absorb any incident waves, regardless of the angle of incidence and wavelength. The low-temperature refractive indices of GaAs and Al${}_{0.9}$Ga${}_{0.1}$As were used in the model of the micropillar [44]. We used the optimal resolutions, which yielded reliable error accuracy of around 3% with a reasonable computation time. The small grid size along the z axis (20 nm) was selected because the light propagates along the z axis with a rapid change in both the optical phase and the amplitude of the micropillar modes. The elliptical micropillar was oriented with the major (minor) axis along x (y) axis. A single broadband dipole source was used to generate optical waves and excite high-order resonant modes. The resonant wavelength was λ${}_{r}$ = 920 nm. The elliptical splitting of the fundamental mode and the refractive index of an anisotropic cavity were used to analyze and optimize the optical and structure parameters of microcavities for further use as single-photon emitters.

The dipoles were located at the antinode of the electric field of the first few excited modes for further classification and determination of the spectral position of higher-order modes for an elliptical micropillar. The properties of the fundamental mode were studied only for a single dipole located at the center of the micropillar.

## 3. Results and Discussions

### 3.1. Optical Modes in Elliptical Microresonators

**Figure 2:**
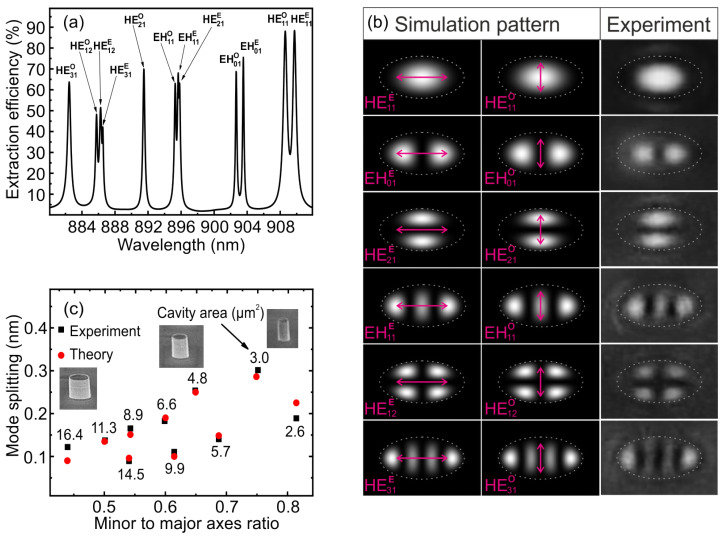
(**a**) Simulated polarized light emission spectrum for several high-order modes of an elliptical micropillar with a major (minor) axis of 2.6 μm (1.3 μm). (**b**) Calculated and measured near-field intensity distribution profiles of the electromagnetic modes of the elliptical microresonator. The arrows show the mode polarization. (**c**) The fundamental mode splitting versus the minor to major axis ratio for elliptical micropillars with a cavity area from 2.6 to 16 μm${}^{2}$ according to experimental data (black symbols) obtained from the measured reflectance spectra and simulation of elliptical micropillars (red symbols) with a cavity anisotropic refractive index.

We considered optical modes in an elliptical micropillar with a major (minor) axis of 2.6 μm (1.3 μm). Reduced symmetry splits earlier twice degenerate fundamental mode HE${}_{11}$ in the cylindrical case into two orthogonally polarized modes (odd and even) in the elliptical microresonator. Elliptical mode classification was performed in terms of Mathieu functions as in [45]. Figure 2a shows the spectral dependence of the extraction efficiency for the fundamental and higher-order optical modes, calculated by the FDTD method. Figure 2b shows the corresponding electric near-field distributions of the optical mode of elliptical resonator with their polarization. The study of the PL of single QDs coupled to elliptical micropillars made it possible to experimentally reveal the existence of various modes. A clear picture of the near field of an elliptical micropillar is observed when the QD emission is spectrally aligned with one of the optical modes. The experimental results are in good agreement with the calculated near-field distributions.

The fundamental mode HE${}_{11}$ clearly splits into two orthogonally polarized modes HE${}_{11}^{O}$ and HE${}_{11}^{E}$ with the highest output efficiency. In addition, the field pattern of the fundamental mode provides the best coupling of luminescence to a single-mode optical fiber. The experimental dependence of the fundamental mode splitting on the minor/major axes ratio is presented in Figure 2c. As expected, an increase in ellipticity as well as a decrease in area leads to an increase in mode splitting. The red dots in Figure 2c show the simulated mode splitting for the respective sizes.

At first, the discrepancy between the simulation and experiment reached 40% when using an isotropic refractive index in the cavity. To eliminate this discrepancy, we took into account the anisotropy of the GaAs refractive index. This anisotropy slightly splits twice degenerate modes into two nondegenerate polarized modes, even in symmetric micropillars. According to the experimental data, the splitting of the fundamental mode in a cylindrical micropillar with a diameter of 3 μm, fabricated as a reference, was about 0.045 nm. To describe the spatially inhomogeneous refractive index in the cavity, we used the diagonal form of the dielectric permittivity tensor ε in the material equations relating the electric displacement field **D** to the electric field strength **E** (**D** = ε**E**). The diagonal components of the permittivity tensor were taken as $\varepsilon_{xx}$ = ($n_{xx}$)${}^{2}$ = 3.5025${}^{2}$, $\varepsilon_{yy}$ = ($n_{yy}$)${}^{2}$ = 3.503${}^{2}$, $\varepsilon_{zz}$ = ($n_{zz}$)${}^{2}$ = 3.503${}^{2}$, and the remaining components of the tensor were taken equal to zero. The difference between the components $\varepsilon_{xx}$ and $\varepsilon_{yy}$ determines the anisotropy of the refractive index for two orthogonal lateral directions due to a small birefringence arising mostly from some residual uniaxial strain in the semiconductor.

Taking into account the anisotropic refractive index allowed us to reduce the discrepancy between the model and experiment for elliptical microresonators that does not exceed 20%. Comparison of the experimental data on the fundamental mode splitting versus the major and minor axis ratio and different elliptical micropillar sizes with the simulation gives good agreement.

**Figure 3:**
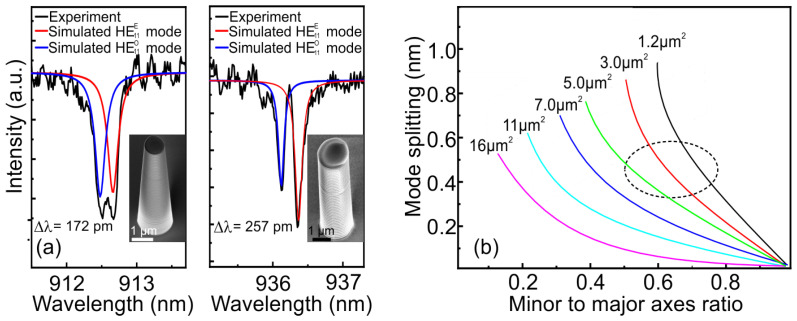
(**a**) Reflectance spectra of elliptical micropillars with a side-wall inclination angle of 3.5 degrees (**left**) and no side-wall angle (**right**). The insets show SEM images of microcavities. (**b**) Simulation of validated fundamental mode splitting curves as a function of minor to major axis ratio for elliptical micropillars with vertical side walls for cavity area from 1.2 to 16 μm${}^{2}$, including anisotropic refractive index.

An important point in the manufacture of an elliptical pillar is the verticality of the side walls. The appearance of even a small convergence angle of the pillar affects not only the optical characteristics of a single-photon emitter but also the magnitude of the mode splitting. Figure 3a shows the experimental (black curves) and simulated (color curves) data of the reflectance spectra for the case of an elliptical microcavity with a side-wall inclination angle of 3.5 degrees (left) and with a vertical side wall (right). The dimensions of the elliptical micropillar with vertical side walls were 3.0 × 1.8 μm, while for the elliptical micropillar with inclined side walls, the dimentions were 2.7 × 1.5 μm at the top and 3.5 × 2.3 μm at the base. It can be seen how the side-wall inclination angle of 3.5 degrees of an elliptical micropillar significantly reduces both the HE${}_{11}^{E}$ and HE${}_{11}^{O}$ modes splitting and the quality factor. For such elliptical micropillars, according to the simulation results, with an increase in the side-walls inclination angle up to 3.5 degrees for the HE${}_{11}^{E}$ mode, the quality factor decreases by 55%. In this case, the mode volume is decreased by 19%, which, as a consequence, leads to a drop in the Purcell factor by 44%. That is why it was so important to fabricate the vertical side walls of elliptical microresonators in the process of dry etching.

Then, several curves were modeled for different sizes with the same pitch of elliptical micropillars with vertical side walls (Figure 3b). Since the volume of the mode increases as the cavity area increases, the Purcell factor is therefore reduced. The pattern of the far field is distorted with an increase in the minor to major axis ratio, thereby reducing the emission to the desired numerical aperture. Thus, the most promising region of potentially reproducible geometric dimensions of elliptical micropillars for resonant excitation is marked with a dotted line. The presence of vertical side walls will provide optimal splitting of the fundamental mode without significant losses in the optical parameters of the microcavity, such as the Purcell factor, output efficiency, and radiation pattern. Accordingly, a model was developed that makes it possible to calculate the splitting of the fundamental mode in elliptical micropillars with good accuracy, taking into account the variation in the size of the ellipses.

### 3.2. QD in Elliptical Microresonator

**Figure 4:**
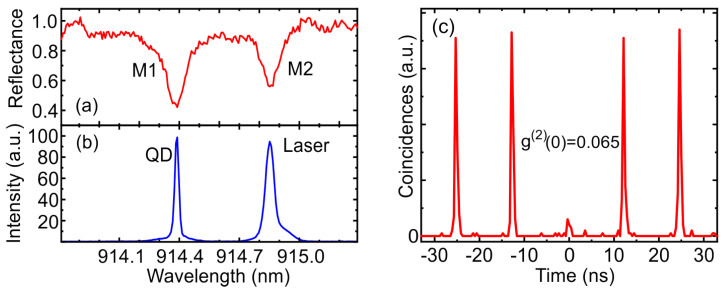
(**a**) Reflectance spectrum of an elliptical microcavity, (**b**) PL spectrum of a QD under resonant excitation and (**c**) the corresponding second-order autocorrelation histogram.

For optical studies, an elliptical micropillar with a major (minor) 2.1 (1.3) μm axis was chosen, demonstrating two non-degenerate fundamental optical modes of the resonator at 914.39 nm (M1) and 914.86 nm (M2) with a quality factor of $Q_{M1}$ = 7500 and $Q_{M2}$ = 8800, respectively (Figure 4a). The emission of a charged QD is spectrally aligned with the short-wavelength mode M1 (Figure 4b), which corresponds to the minor axis of the microcavity. According to FDTD calculations, the fundamental optical mode is closer to the side wall of the minor axis of the elliptical micropillar and therefore has a lower quality factor due to the occurrence of losses arising from the side-wall roughness [16]. However, the fundamental linearly polarized optical cavity M1 mode has a smaller mode volume, which leads to an increase in the Purcell factor and the M1 mode output efficiency. Thus, the use of the short-wavelength mode is one of the factors for obtaining a high efficiency of a SPS in an elliptical microcavity.

The QD was optically excited by a mode-locked Ti:sapphire laser tuned to the M2 mode. The cross-polarization method was used to suppress the backscattered laser. The FWHM of the laser pulse, reduced by a pulse shaper [46], was chosen in such a way as to match the microcavity mode and provide polarization filtering. The best filtering was achieved with a laser FWHM of 55 pm, which is rather narrow for purely resonant excitation. However, the QD still exhibits a bright luminescence, which is probably due to the appearance of an additional excitation channel associated with the interaction with acoustic phonons.

**Figure 5:**
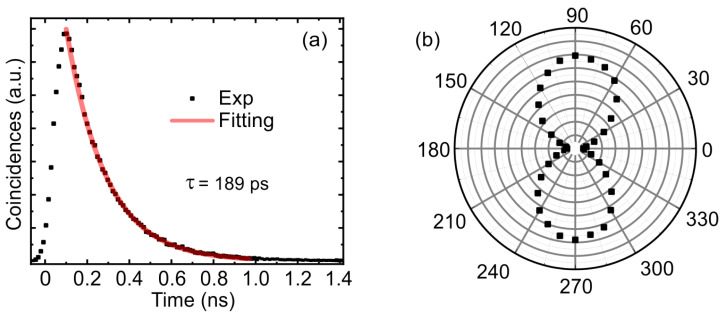
(**a**) Time resolved PL measurement of a charged QD under resonant excitation. (**b**) Polarization-resolved PL measurement of the QD under non-resonant excitation conditions.

The second-order correlation function measured in the Hanbury Brown and Twiss setup demonstrates the single-photon behavior of the emitted QD with g${}^{\left(2\right)}\left(0\right)$ = 0.065 (Figure 4c). The imperfection of the measured value of g${}^{\left(2\right)}\left(0\right)$ is mainly connected with the incomplete filtering of laser radiation due to the higher excitation power compared to direct resonant excitation. The time-resolved PL measurement demonstrates a strictly exponential decay curve corresponding to the recombination of the trion state with a lifetime of 189 ps (Figure 5a). Since the average lifetime of trion states in a bulk material is about 1 ns ([47]), the Purcell factor in the corresponding M1 optical mode $F_{M1}$ can be taken to be approximately equal to 5.

The rate of QD transition in an elliptical microcavity will be accelerated differently at different polarizations, which depends on the cavity linewidth δω and frequency separation Δω [40]: $F_{M1}$*⁄*$F_{M2}$ = 1 + 4(Δω*⁄*δ$\omega_{M1}$). In our experiment, the ratio Δω*⁄*δ$\omega_{M1}$ is equal to 3.9, that leads to a 62 times smaller Purcell factor in the orthogonal M2 mode for the selected QD. The QD spontaneous emission efficiency depends on the Purcell factor and the corresponding quality factor Q [48]: η = *F⁄*(*F* + 1)×*Q*⁄$Q_{0}$, where $Q_{0}$ is a quality factor of planar cavity. According to this formula, a QD with a probability of 90.2% (defined as $\eta_{M1}$*⁄*($\eta_{M1}$ + $\eta_{M2}$)) emits in the M1 mode, and the luminescence of charged QD in the asymmetric microresonator turns out to be highly polarized.

To experimentally determine the emission probability in the M1 mode, the PL signal was measured as a function of the rotation angle of the detection polarization (Figure 5b). To avoid effects associated with polarization orientation, above-barrier excitation was used. According to the experimental data, the probability of QD emission in the short-wavelength M1 mode is 91.2%, which is in a good agreement with the estimated value.

## 4. Conclusions

In this paper, we analyzed the impact of the ellipticity, ellipse area, and verticality of the side walls on the splitting of the fundamental optical mode, both theoretically and experimentally. We managed to achieve good agreement between the experimental data and the theoretically modeled data, taking into account the small anisotropy of the refractive index of GaAs. This made it possible to reliably calculate potential design options for elliptical microcavities to obtain the required mode splitting. The critical sensitivity of these systems to the verticality of the microresonator side walls is revealed. The study of the near-field pattern allowed us to detect the presence of higher-order optical modes, which were classified theoretically. Highly polarized single-photon QD emission associated with the short-wavelength fundamental cavity mode was demonstrated. This investigation opens the way to the possibility of further increasing the brightness of SPSs.

## Data Availability

The data that support the findings of this study are available within the article.

## References

[1] Knill E., Laflamme R., Milburn G.J. (2001). A scheme for efficient quantum computation with linear optics. Nature.

[2] Wang H., He Y.-M., Li Y.-H., Su Z.E., Li B., Huang H.L., Ding X., Chen M.C., Liu C., Qin J. (2017). High-efficiency multiphoton boson sampling. Nat. Photonics.

[3] Bouwmeester D., Pan J.W., Mattle K., Eibl M., Weinfurter H., Zeilinger A. (1997). Experimental quantum teleportation. Nature.

[4] Zhao J., Ma C., Rüsing M., Mookherjea S. (2020). High quality entangled photon pair generation in periodically poled thin-film lithium niobate waveguides. Phys. Rev. Lett..

[5] Ma C., Wang X., Anant V., Beyer A.D., Shaw M.D., Mookherjea S. (2017). Silicon photonic entangled photon-pair and heralded single photon generation with CAR > 12000 and g^2^(0) < 0.006. Opt. Express.

[6] Michler P., Kiraz A., Schoenfeld W.V., Petroff P.M., Zhang L., Hu E., Imamoglu A. (2000). A quantum dot single-photon turnstile device. Science.

[7] He Y.-M., He Y., Wey Y.-J., Wu D., Atature M., Schneider C., Hofling S., Kamp M., Lu C.-Y., Pan J.-W. (2013). On-demand semiconductor single-photon source with nearunity indistinguishability. Nat. Nanotechnol..

[8] Beugnon J., Jones M.P., Dingjan J., Darquié B., Messin G., Browaeys A., Grangier P. (2006). Quantum interference between two single photons emitted by independently trapped atoms. Nature.

[9] Higginbottom D.B., Slodička L., Araneda G., Lachman L., Filip R., Hennrich M., Blatt R. (2016). Pure single photons from a trapped atom source. New J. Phys..

[10] Maunz P., Moehring D., Olmschenk S., Younge K.C., Matsukevich D.N., Monroe C. (2007). Quantum interference of photon pairs from two remote trapped atomic ions. Nat. Phys..

[11] Zhang Q., Dang C., Urabe H., Wang J., Sun S., Nurmikko A. (2008). Large ordered arrays of single photon sources based on II-VI semiconductor colloidal quantum dot. Opt. Express.

[12] Aharonovich I., Englund D., Toth M. (2016). Solid-state single-photon emitters. Nat. Photonics.

[13] Bernien H., Childress L., Robledo L., Markham M., Twitchen D., Hanson R. (2012). Two-photon quantum interference from separate nitrogen vacancy centers in diamond. Phys. Rev. Lett..

[14] Holmes M.J., Choi K., Kako S., Arita M., Arakawa Y. (2014). Room-temperature triggered single photon emission from a III-nitride site-controlled nanowire quantum dot. Nano Lett..

[15] Rakhlin M., Sorokin S., Kazanov D., Sedova I., Shubina T., Ivanov S., Mikhailovskii V., Toropov A. (2021). Bright single-photon emitters with a CdSe quantum dot and multimode tapered nanoantenna for the visible spectral range. Nanomaterials.

[16] Gazzano O., Michaelis de Vasconcellos S., Arnold C., Nowak A., Galopin E., Sagnes I., Lanco L., Lemaître A., Senellart P. (2013). Bright solid-state sources of indistinguishable single photons. Nat. Commun..

[17] Friedler I., Sauvan C., Hugonin J.P., Lalanne P., Claudon J., Gerard J.-M. (2009). Solid-state single photon sources: The nanowire antenna. Opt. Express.

[18] Claudon J., Bleuse J., Malik N.S., Bazin M., Jaffrennou P., Gregersen N., Sauvan C., Lalanne P., Gérard J.-M. (2010). A highly efficient single-photon source based on a quantum dot in a photonic nanowire. Nat. Photonics.

[19] Rakhlin M., Klimko G., Sorokin S., Kulagina M., Zadiranov Y., Kazanov D., Shubina T., Ivanov S., Toropov A. (2022). Bright single-photon sources for the telecommunication O-band based on an InAs quantum dot with (In)GaAs asymmetric barriers in a photonic nanoantenna. Nanomaterials.

[20] Kuhlmann A., Houel J., Ludwig A., Greuter L., Reuter D., Wieck A.D., Poggio M., Warburton R.J. (2013). Charge noise and spin noise in a semiconductor quantum device. Nat. Phys..

[21] Purcell E.M., Torrey H.C., Pound R.V. (1946). Resonance Absorption by Nuclear Magnetic Moments in a Solid. Phys. Rev..

[22] Madsen K.H., Ates S., Liu J., Javadi A., Albrecht S.M., Yeo I., Stobbe S., Lodahl P. (2014). Efficient out-coupling of high-purity single photons from a coherent quantum dot in a photonic-crystal cavity. Phys. Rev. B.

[23] Arcari M., Söllner I., Javadi A., Albrecht S.M., Yeo I., Stobbe S., Lodahl P. (2014). Near-unity coupling efficiency of a quantum emitter to a photonic crystal waveguide. Phys. Rev. Lett..

[24] Wang H., Hu H., Chung T.-H., Qin J., Yang X., Li J.-P., Liu R.-Z., Zhong H.-S., He Y.-M., Ding X. (2019). On-demand demiconductor source of entangled photons which simultaneously has high fidelity, efficiency, and indistinguishability. Phys. Rev. Lett..

[25] Santori C., Fattal D., Vučković J., Solomon G.S., Yamamoto Y. (2002). Indistinguishable photons from a single-photon device. Nature.

[26] Ding X., He Y., Duan Z.-C., Gregersen N., Chen M.-C., Unsleber S., Maier S., Schneider C., Kamp M., Höfling S. (2016). On-demand single photons with high extraction efficiency and near-unity indistinguishability from a resonantly driven quantum dot in a micropillar. Phys. Rev. Lett..

[27] Somaschi N., Giesz V., De Santis L., Loredo J.C., Almeida M.P., Hornecker G., Portalupi S.L., Grange T., Antón C., Demory J. (2016). Near-optimal single-photon sources in the solid state. Nat. Photonics.

[28] Ollivier H., Maillette de Buy Wenniger I., Thomas S., Wein S.C., Harouri A., Coppola G., Hilaire P., Millet C., Lemaître A., Sagnes I. (2020). Reproducibility of high performance quantum dot single photon sources. ACS Photonics.

[29] Tomm N., Javadi A., Antoniadis N.O., Najer D., Löbl M.C., Korsch A.R., Schott R., Valentin S.R., Wieck A.D., Ludwig A. (2021). A bright and fast source of coherent single photons. Nat. Nanotechnol..

[30] Rakhlin M.V., Galimov A.I., Dyakonov I.V., Skryabin N., Klimko G., Kulagina M., Zadiranov Y., Sorokin S., Sedova I., Guseva Y. (2023). Demultiplexed single-photon source with a quantum dot coupled to microresonator. J. Lumin..

[31] He Y.-M., Wang H., Wang C., Chen M.-C., Ding X., Qin J., Duan Z.-C., Chen S., Li J.-P., Liu R.-Z. (2019). Coherently driving a single quantum two-level system with dichromatic laser pulses. Nat. Phys..

[32] Thomas S.E., Billard M., Coste N., Wein S.C., Priya, Ollivier H., Krebs O., Tazaïrt L., Harouri A., Lemaitre A. (2021). Bright polarized single-photon source based on a linear dipole. Phys. Rev. Lett..

[33] Ortiz O., Pastier F., Rodriguez A., Priya, Lemaitre A., Gomez-Carbonell C., Sagnes I., Harouri A., Senellart P., Giesz V. (2020). Fiber-integrated microcavities for efficient generation of coherent acoustic phonons. Appl. Phys. Lett..

[34] Gayral B., Gérard J.M., Legrand B., Costard E., Thierry-Mieg V. (1998). Optical study of GaAs/AlAs pillar microcavities with elliptical cross section. Appl. Phys. Lett..

[35] Unitt D.C., Bennett A.J., Atkinson P., Ritchie D.A., Shields A.J. (2005). Polarization control of quantum dot single-photon sources via a dipole-dependent Purcell effect. Phys. Rev. B.

[36] Lee Y., Lin S. (2014). Polarized emission of quantum dots in microcavity and anisotropic Purcell factors. Opt. Express.

[37] Uğur Meriç G., Michael M., Samel A., Niels G. (2021). Elliptical micropillar cavity design for highly efficient polarized emission of single photons. Appl. Phys. Lett..

[38] Daraei A., Tahraoui A., Sanvitto D., Timpson J.A., Fry P.W., Hopkinson M., Guimarães P.S.S., Vinck H., Whittaker D.M., Skolnick M.S. (2006). Control of polarized single quantum dot emission in high-quality-factor microcavity pillars. Appl. Phys. Lett..

[39] Strauf S., Stoltz N., Rakher M., Coldren L.A., Petroff P.M., Bouwmeester D. (2007). High-frequency single-photon source with polarization control. Nat. Photonics.

[40] Wang H., He Y.-M., Chung T.-H., Hu H., Yu Y., Chen S., Ding X., Chen M.-C., Qin J., Yang X. (2019). Towards optimal single-photon sources from polarized microcavities. Nat. Photonics.

[41] Varoutsis S., Laurent S., Sagnes I., Lemaître A., Ferlazzo L., Mériadec C., Patriarche G., Robert-Philip I., Abram I. (2005). Reactive-ion etching of high-Q and submicron-diameter GaAs/AlAs micropillar cavities. J. Vac. Sci. Technol..

[42] Heuser T., Grobe J., Holzinger S., Sommer M., Reitzenstein S. (2020). Development of highly homogenous quantum dot micropillar arrays for optical reservoir computing. IEEE J. Sel. Top. Quantum Electron..

[43] Joint F., Abadie C., Vigneron P.B., Boulley L., Bayle F., Isac N., Cavanna A., Cambril E., Herth E. (2020). GaAs manufacturing processes conditions for micro- and nanoscale devices. J. Manuf. Process..

[44] Bobrov M.A., Blokhin S.A., Maleev N.A., Kuz’menkov A.G., Vasil’ev A.P., Guseva Y.A., Rakhlin M.V., Galimov A.I., Serov Y.M., Troshkov S.I. (2022). Simulation and analysis of the optical characteristics of cylindrical micropillars with InAs/GaAs quantum dots. JETP Lett..

[45] Yeh C., Shimabukuro F.I. (2010). The Essence of Dielectric Waveguides.

[46] Monmayrant A., Weber S., Chatel B. (2010). A newcomer’s guide to ultrashort pulse shaping and characterization. J. Phys. B At. Mol. Opt. Phys.

[47] Wang G., Fafard S., Leonard D., Bowers J.E., Merz J.L., Petroff P.M. (1994). Time-resolved optical characterization of InGaAs/GaAs quantum dots. Appl. Phys. Lett..

[48] Barnes W., Björk G., Gérard J., Jonsson P., Wasey J.A.E., Worthing P.T., Zwiller V. (2002). Solid-state single photon sources: Light collection strategies. Eur. Phys. J. D.

